# Implementing Virtual Simulated Person Methodology to Support the Shift to Online Learning: Technical Report

**DOI:** 10.7759/cureus.8864

**Published:** 2020-06-27

**Authors:** Eva Peisachovich, Celina Da Silva, Nathaniel J Penhearow, Elizabeth V Sombilon, Marianne Koh

**Affiliations:** 1 Medical Education and Simulation, York University, Toronto, CAN; 2 Cardiology, North York General, Toronto, CAN; 3 Nursing, York University, Toronto, CAN; 4 Faculty of Health, York University, Toronto, CAN

**Keywords:** simulated persons, virtual workshop, remote learning

## Abstract

The COVID-19 pandemic has dramatically changed how education is delivered worldwide. The resultant rise of e-learning, whereby teaching is undertaken remotely and on digital platforms, has extensively impacted universities and other higher education organizations around the world. One approach to support this change in education delivery is the use of virtual simulation approaches. Our team at SimXSpace has piloted a virtual workshop using Zoom, an online video-conferencing platform, and virtual simulated persons (SPs) to support communication and interpersonal skills among learners.

The main objective of the pilot virtual workshop was to develop and implement the SP methodology remotely via the Zoom platform (Zoom Video Communications, San Jose, California) and to evaluate its effectiveness as an immersive environment for simulation. The virtual workshop involved four instructors who intend to implement virtual SPs within their courses, two workshop facilitators, and two SPs. The workshop was conducted synchronously using Zoom features.

The workshop followed a predefined structure and was completed as planned. Outcomes suggest that remote simulation delivery using virtual SPs and delivered online via Zoom is feasible and provides an effective environment in which to conduct SP methodology to teach communication and interpersonal skills. The findings suggest that remote simulation and virtual SPs can support experiential education and provide an effective and engaging learning environment.

The virtual workshop was successful and laid a foundation for future online training programs for the use of SP methodology. Moreover, it formed an effective outline for subsequent iterations of this virtual training workshop and prompted discussion of plans for future workshops with various programs across a pan-university context.

## Introduction

Due to the COVID-19 pandemic, educational systems in Canada are under increasing pressure to use innovative, creative ways to transform education virtually while increasing critical thinking and reasoning skills among learners. Moreover, the rapid increase of COVID-19 cases worldwide has led universities to cancel workshops, conferences, and other activities for an indefinite period of time. As universities transition to online and remote learning, it becomes increasingly critical to introduce and create innovative virtual-learning opportunities to support learner success and graduate transition to the practice setting and workplace. Moreover, the shift to online interactions has increased the need for telecommunication and teleconsultation; it is, therefore, essential to develop communication and interpersonal skills through this medium in order to support the increasing need for these interactions.

Simulation-training methodology - broadly, a technique that attempts to recreate characteristics of the real world - is one suggested strategy for enhancing critical-thinking, decision-making, problem-solving, and crisis-management skills [1,2]. Its learner-centred, experiential-education approach allows educators to create a meaningful environment for active and interactive learning by introducing realistic scenarios to meet learning objectives [3-6]. These scenarios provide a bridge between theory and practice, as students gain practical experience, immediate feedback, the opportunity to enhance the integration of theory and practice through debriefing and guided reflection, and the ability to apply knowledge and skills in a safe environment [1,2]. Research identifies simulation as an approach that supports the synthesis of knowledge and the development of insight. It is, therefore, essential that teaching institutions take a participatory and collaborative approach in the application, development, and use of this methodology for training novice professionals [1,2,7].

Simulated-person (SP) methodology is a type of simulation that utilizes individuals trained to play roles in specific scenarios designed to meet learning objectives and provide realistic practice. An SP is trained to realistically reproduce scenarios by providing specific information, displaying signs and behaviours, and creating a realistic encounter in a consistent manner [7]. In follow-up debriefings, SPs are trained to provide learners with feedback about their professional manner, attitude, and interpersonal skills, thus promoting individualized, rather than standardized, learning; this feedback is immediate and from the “person’s” point of view. 

Further, to succeed in a given practice setting, the following are required: professionalism, teamwork and interprofessional communication, and collaboration [8]. Applying the use of SPs as a simulation approach aids learners in achieving these objectives by allowing students to learn and practice interpersonal and interprofessional communication skills through meaningful, realistic, human encounters followed by guided reflection in a safe setting.

The use of SPs in clinical education provides many advantages: the instructor-developed scenarios can be repeated to provide a comparable experience for all students; the methodology provides unique opportunities for teaching and learning and for evaluating clinical skills; moreover, it is popular with learners. An encounter using SPs provides a unique approach to enhance teaching strategies related to communication and interpersonal skills, as SPs provide learners with feedback about their mannerisms, body language, depth of explanation of crucial areas, and approaches to sensitive content during the encounter [9].

Hence, our team at SimXSpace has adopted an alternative approach to provide simulation through the use of virtual SPs and to extend the application of simulation across a number of disciplines and beyond the higher education milieu, where it is currently underutilized. Our first pilot workshop using virtual SPs and Zoom (Zoom Video Communications, San Jose, California) was conducted in collaboration with the Health Leadership and Learning Network (HLLN) at York University. The HLLN, which offers continuing education for health professionals, integrates SPs into their coursework for formative learning and summative evaluations.

## Technical report

The purpose of the virtual workshop was to field test the remote use of SP methodology and put the SP’s “role on its feet” to ensure the scenario works when it is “off the page.” The workshop delivery objectives were critical to the process in order to (a) ensure that the scenarios supported the learner’s learning needs, thus allowing them to demonstrate learning objectives; and (b) develop the SP role in a manner that ensures the scenario’s focus. The workshop followed a predefined structure and was completed as planned. Outcomes suggest that remote simulation delivery using virtual SPs through Zoom online is a feasible and effective means to support the development of communication and interpersonal skills. Our findings suggest that remote simulation and virtual SPs can support experiential education and provide an effective and engaging learning environment.

Zoom features

While there are many video-conferencing platforms currently available, including Microsoft Teams, some platforms do not offer screen recording for future reflection, and/or pose accessibility issues for users without an Outlook email address [10]. We chose to use Zoom because of its familiarity, availability, and screen recording features, and because free attendee accounts can easily be created.

**Figure 1:**
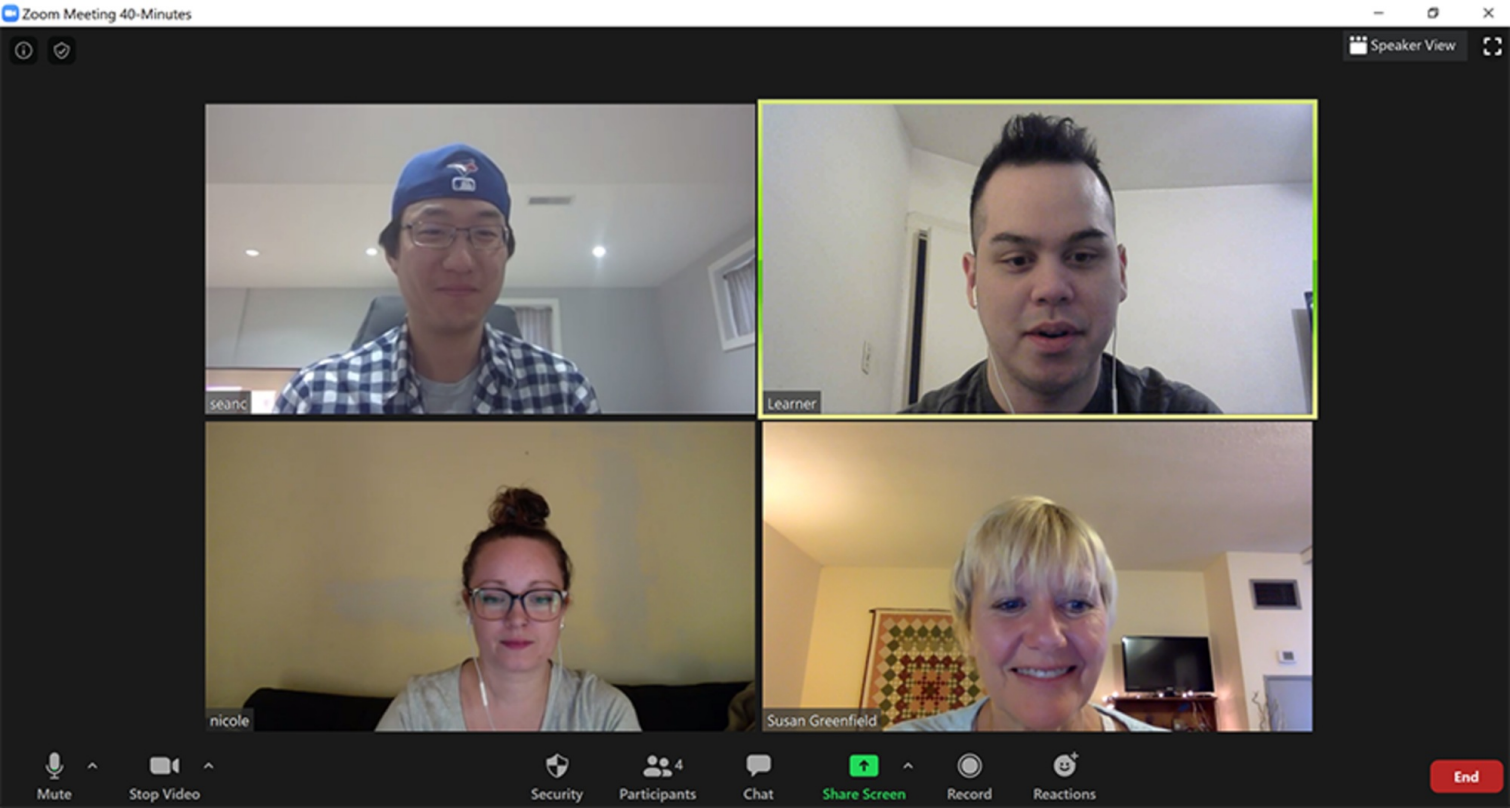
Video-conferencing platform during open collaboration

**Figure 2:**
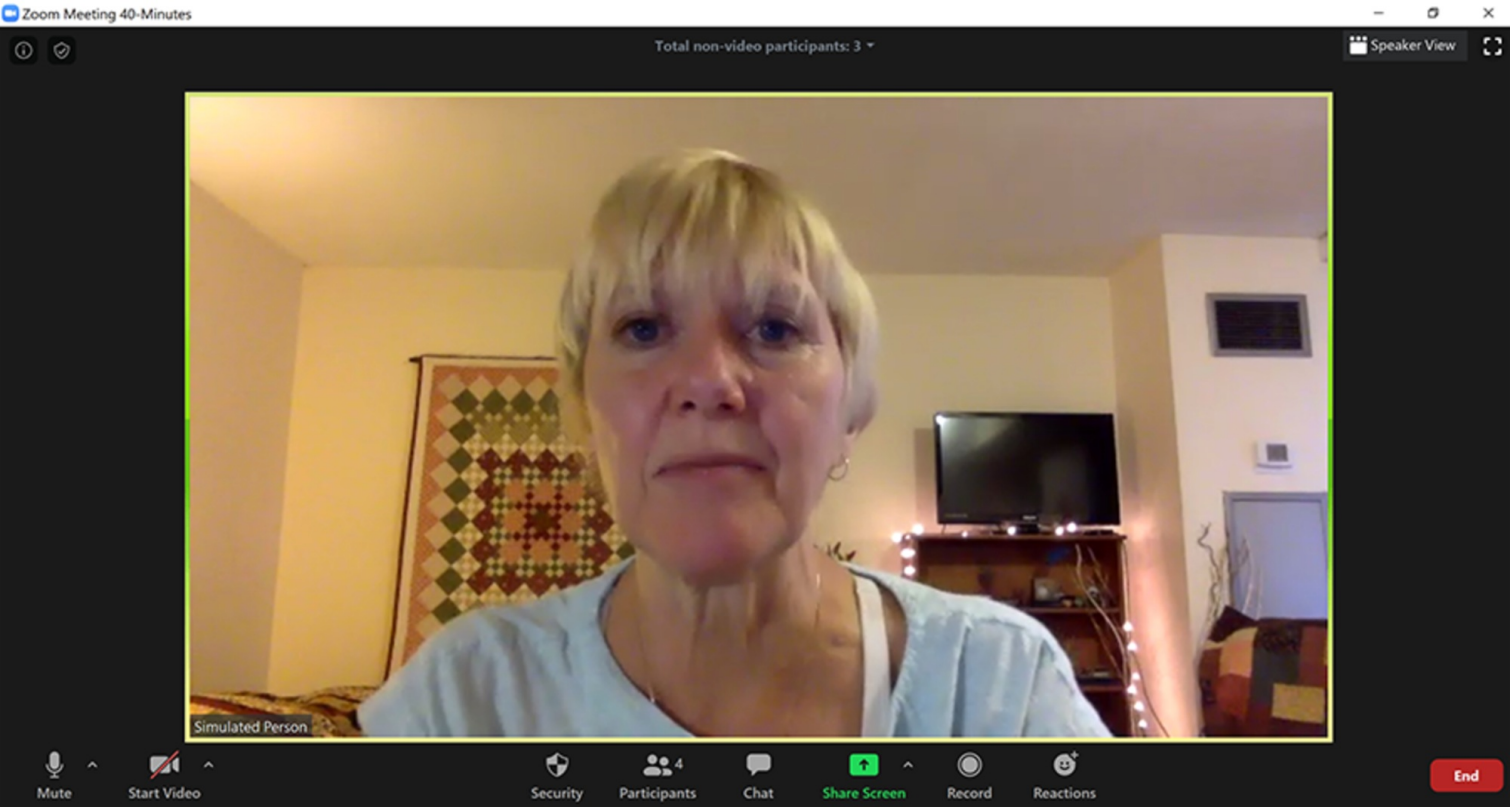
Speaker view used during simulation

Zoom is a video-conferencing platform capable of hosting up to 300 participants at one time. There is no financial commitment for participants, and it is free to download. We used a “Pro” Zoom account, opting to pay a subscription fee to unlock recording features, and remove the 45-minute time limit. To join a meeting, attendees must enter an automatically generated meeting password, which can be sent out prior to the meeting, and be let in by the host (this requirement helps to prevent unwanted sharing.) Attendees can join using a variety of devices, over all major platforms and operating systems. In the pilot workshop, the use of the text-chat features was encouraged to help distil thoughts and limit the number of participants speaking at one time. During simulation, the chat feature can be hidden by those participating in the simulation, allowing those observing to capture and share key insights without disrupting the simulation. Different views of video feeds allowed the facilitation of a more natural collaborative, or “one-on-one,” interaction, as illustrated in Figures 1 and 2.

Other features included the use of reactions, including a “thumbs up” or “thumbs down,” which enabled attendees to quickly vote on the next step that should be taken in a given scenario. Similarly, participants could “raise a hand,” allowing a more autocratic style when required. Establishing ground rules and best-practice guidelines is recommended at the outset of the virtual workshop. Using headsets with microphones and muting one’s own mic are encouraged to limit environmental noise and unnecessary speech. The sharing of videos was encouraged during collaborative sections, as this encourages engagement and provides visuals to augment conversation. Participants were asked to ensure that their head and shoulders were in clear view and were encouraged to work in a quiet environment with even lighting. Reliable access to the internet was required to prevent connection issues. The recording feature was used during the workshop; this allowed participants to view the simulation interactions after the workshop for further reflection and refinement.

Virtual workshop structure

The group consisted of four instructors: two facilitators and two SPs. Instructor participants worked to develop scenarios and facilitate interactions with diverse learners, allowing them to observe how the scenarios were actualized as a teaching tool in practice. One facilitator’s strong technical background helped orient other participants to the Zoom interface and provided ad hoc technical support. Furthermore, administrative support by facilitators ensured that the scenario template was updated collaboratively in a word-processing application in real time, and ideas posted in the chat function were captured and followed up on.

**Figure 3:**
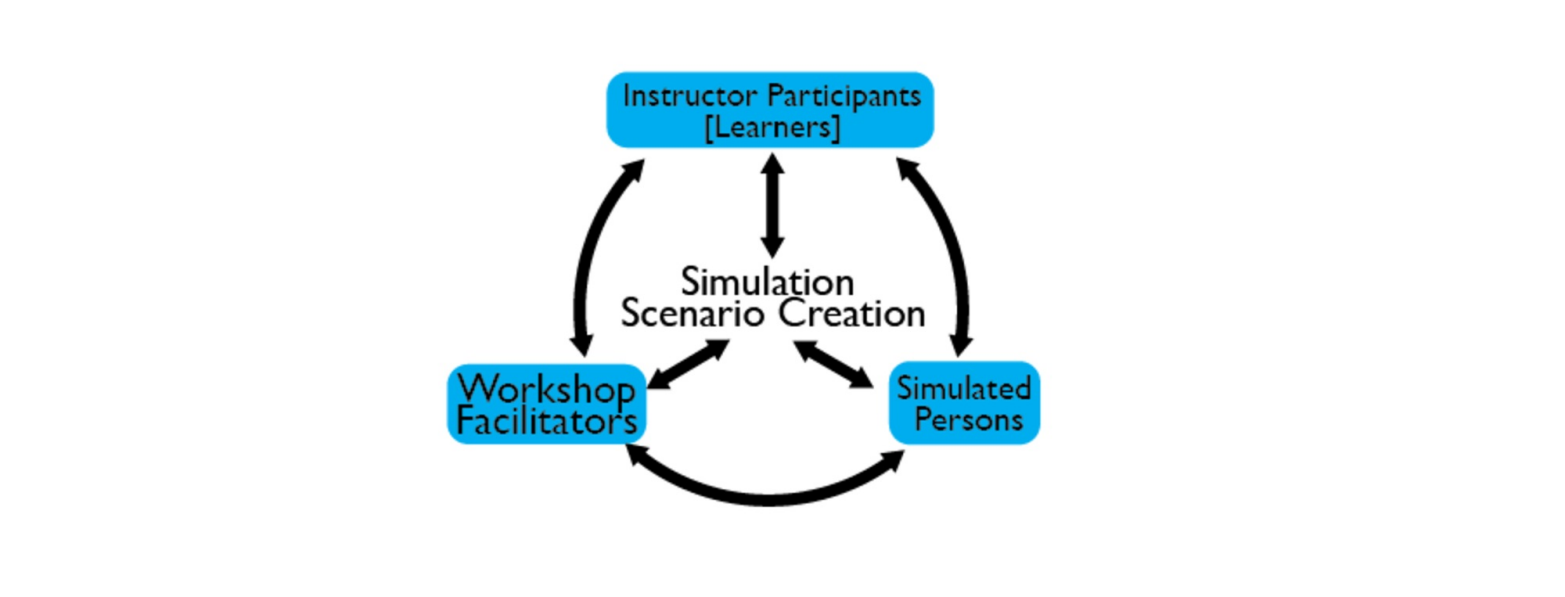
Workshop design used to develop simulation scenario

The objectives of the pilot workshop were to (a) provide instructor participants an opportunity to engage with SPs as learners and to experience the simulation scenario through a learner’s lens; (b) provide the SPs an opportunity to simulate each role with different learners; (c) provide instructor participants with a deeper understanding of the simulation process, including debrief and facilitation processes; and (d) provide the SPs an opportunity to provide feedback about the learner interaction (Figure 3).

In total, four hours were spent on activities in a large group (see Table 1).

**Table 1:** Schedule of simulated person methodology virtual workshop

Workshop Timeframe	Workshop Activities
10 minutes	Ensure workshop participants are virtually connected + review of ground rules
10 minutes	Introductions, welcome, and prebriefing
20 minutes	Demonstrate three different simulated-person encounter variations with time-outs
60 minutes	Review objectives for the simulation scenarios + field test roles and scenarios
20 minutes	Breaktime
90 minutes	Put scenarios on their feet using simulated persons, with instructor participants functioning as the learners
30 minutes	Debrief and wrap-up

In order to align with the simulated-scenario learning objectives, both the SP-methodology scenarios and feedback-model templates were provided prior to the workshop as a helpful resource to guide scenario development; all workshop participants were prepared beforehand (Appendices 1 and 2).

**Figure 4:**
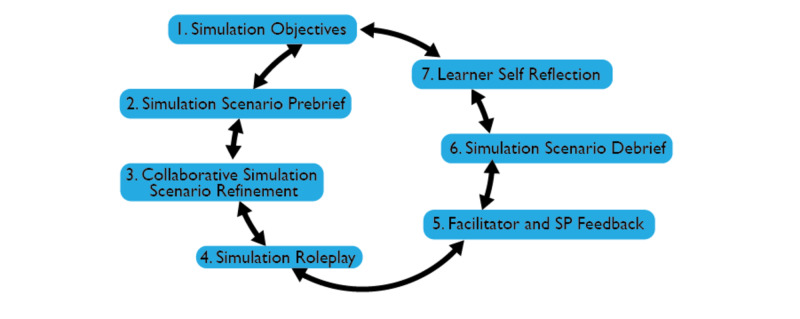
Workshop format to deliver online simulation

As a group of educators with a breadth of experience in simulation methodology, we used the model of appreciative inquiry as a method [11]. This method was threaded through the workshop and allowed us both to explore and investigate the strengths of simulation and SP methodology as an experiential tool and to develop a design that could be applied in a multi-disciplinary environment, as illustrated in Figure 4.

When putting the “scenario on its feet,” only the instructor participant who created the scenario and the SP were able to use the screen; other workshop participants were asked to stop their videos and hide all non-video participants while the SP and the scenario developer or instructor participant engaged in simulation with one-to-one video. This enhanced immersion minimized distractions of self-observation and maximized the opportunity to observe non-verbal mannerisms. This also afforded an opportunity to experience being an observer. 

The use of the chat feature supported other participants or observers in providing feedback and engaging in the debrief process. The opportunity for instructor participants to participate in the simulation as learners and facilitators served to both inform scenario development and elicit feedback from various perspectives. Recordings of the practice simulation were provided to educators after the workshop for further scenario development and self-reflection.

## Discussion

The pilot virtual workshop builds on the impact of SP methodology by investigating virtual SP applications using the Zoom online environment; by evaluating the virtual workshop’s efficacy as an experiential-education tool, the pilot provides a fuller understanding of the effectiveness of SP methodology from a multi-disciplinary perspective and of its influence on professional development beyond the allied-health lens and disciplines.

The use of virtual SPs should involve premeditation regarding how this learning experience will enhance theory-praxis understanding; this workshop was a starting point for this ongoing project. There is a large research focus, across the health professions, on clinical-skill acquisition as a learning objective of simulation. Yet simulation activities can be also utilized to develop competence in communication, professionalism, cultural sensitivity, ethics, and many other applications, all of which receive less research emphasis, particularly in professions outside of healthcare [2].

Given the importance of evaluating the level of authenticity required for the simulation to engage learning and to suspend disbelief, the process of developing simulation scenarios requires thoughtful engagement informed by sound pedagogical frameworks to support robust learning opportunities [12]. To ensure that these important elements of simulation-based education were honoured in our project, we chose to begin our pilot with the HLLN, as this department is already somewhat familiar with simulation pedagogy and the nature of authentic case-based learning [13]. As a continuing education unit for healthcare professionals, the HLLN needs to use teaching methods that are practical and applied for working professionals, with relevance to their workplaces. HLLN planned to develop summative assessments for two of its courses: Health Coaching and Patient Navigation. As electronic healthcare (e-health) delivery continues to gain traction, especially given the COVID-19 pandemic, HLLN set out to build the summative assessments using a virtual experiential-learning method to mirror e-health delivery. With funding from eCampus Ontario - an organization with a mandate to promote access, collaboration, and innovation in online and technology-enabled learning in Ontario - HLLN worked with the SimXSpace team to develop and test four simulated case scenarios. The simulations created during the workshops will be used both in small breakout group practice during the HLLN courses and for individual summative assessments. In the summative assessment, learners will be expected to complete three 20-minute simulation sessions; one of these three sessions will be observed by the instructor and graded using a scoring rubric.

The workshop resulted in an increase in participants’ knowledge about the application of SP methodology in the teaching-learning context and offered insight into experiential-education innovation using virtual SPs, yet there is limited evidence regarding a similar improvement in communication and interpersonal skills. Given the lack of studies conducted within higher education milieus and in comparable populations, further research is needed to determine how the application of virtual SPs can enhance and sustain learners’ communication and interpersonal skills and instructors’ teaching and learning skills. Further research is also needed to explore the use of this version of the workshop with faculty teaching other disciplines and among different learner cohorts.

The foremost goal of simulation as a pedagogy is to enhance learning; however, cost-effectiveness is crucial if this educational technique is to be seen as a viable option for institutions [14]. There is a noticeable challenge regarding both cost-effectiveness and the evaluation of the investment return of simulation education [14-16]. The virtual delivery of SPs has been shown to also be more cost effective, which may enhance its use within higher education milieus. Further, the online milieu provides a more feasible process for designing, developing, and producing instructional materials for the first offering, not to mention that it is more accessible. The one-time technological infrastructure costs of virtual simulation are offset, as it requires no travel accommodations, and physical space costs are incurred for neither simulation training nor classrooms in which to conduct the simulation. Individual organizations often attempt to decrease costs of simulation through anecdotal approaches or by using less expensive means to meet their particular educational needs [16]. Hence, using virtual SPs may support the attempt to decrease costs and close the gap in the body of evidence to demonstrate the efficiency of simulated-learning approaches [15,16].

In general, the workshop was successful and laid a foundation for future training programs of this style and allowed our team to further refine the workshop to meet the needs of educators looking to embed SP methodology in their curricula. The workshop attendees were satisfied, as evidenced by feedback garnered through an open-ended questionnaire that indicated increased confidence with delivering SP methodology remotely and facilitating learners’ learning experience through this means. 

Our experience with this workshop will allow us to design a blueprint for a cross-disciplinary program to provide educators with training in simulation methodology and undergraduate students with an opportunity to practice and develop competencies in their respective fields. Future planning is underway to further develop and refine the work begun with this workshop.

Limitations

The virtual workshop laid a foundation for future online-training programs of this style, as it provides an effective outline for subsequent iterations; plans for future workshops are in discussion. Yet it also identified some limitations to be considered, regarding the use of body language and non-verbal skills. Technical limitations were also observed with regards to internet-connection speed and reliability.

Traditionally, communication “off-line” relies on both parties being in a room together. There are many complicated and subliminal cues in body language that contribute to the interactive nature of communication. In a virtual simulation, however, the view of a participant is limited by the field of view of that participant’s webcam. Therefore, certain body-language habits that are effective in face-to-face communication may not be transmitted or may go unnoticed in virtual simulation. For example, participants may lean in, demonstrating interest and engagement, but this gesture may move outside of the camera’s field of view. The attention given to how the SP’s camera is presented sets the upper limits of what non-verbal cues can be picked up on. Having the SP wear clothes that contrast with the background facilitates body positioning, and positioning the camera the farther back, or using a wider lens, will allow more of the communicator’s body to be viewed.

Furthermore, not all video devices are ideally located close to the eye-line, which approximates but does not replace eye contact. To establish eye contact, the participant must look directly into the lens of the webcam, not into the other participant’s eyes on the screen. Looking into the lens facilitates the feeling of engagement for the receiver of the video, but disrupts the engagement of the caster. This effect is more clearly observed with technology where built-in webcams are located between the keyboard and screen, or are off-centre.

We also acknowledge the possibility that access to a stable internet connection can be a limiting factor. An unexpected drop in the video feed could break the immersive experience of the simulation. If the participants are emotionally engaged and the simulation and support suddenly stop, it will prevent timely debriefing, impede the immersive factor, and create frustration among users. 

Recommendations and next steps

The importance of engaging learners underscores the value of the SP methodology approach, as learners interact through scenarios that simulate the realities and complexities of practice-realities that often do not match the textbook portrayal; thus, SP methodology contributes to learners’ success in their transition to the workplace milieu. Creating opportunities to enhance online engagement through the use of simulation provides a promising direction to bring experiential education into the online context; in light of the COVID-19 outbreak and in preparation for potential future outbreaks, the provision of such opportunities becomes critical in higher education contexts. We intend to pilot the virtual SP methodology within various disciplines and contexts-from nursing and social work, where these virtual simulations can replace clinical-placement opportunities that require patient consultation, to fields such as education and museum studies, where the simulation can help learners acquire communication and interpersonal skills.

Although it was not identified through this pilot workshop, when shifting to virtual SP encounters it is useful to consider the challenges and limitations that may be faced by the learners, instructors, and SPs involving aspects related to professional competence, professionalism, and non-verbal communication skills. By adjusting virtual encounters to respond to these challenges, educators can support the implementation of online simulation encounters and training, embed opportunities to teach communication skills and the affective domain, and provide guidance with curriculum design when using online approaches-even post COVID-19, as telecommunication and tele-consultations will become much more common.

Although our pilot project identified how the use of virtual SPs can support communication and interpersonal skills among learners, we realize that within healthcare education there is a need to develop ways to conduct physical examinations virtually, as learners become frustrated with the inability to perform physical examinations [17]. Further, we recommend that, in the event that simulations end abruptly due to technological breakdown, a backup form of communication or an administrator be available to re-establish contact in a timely manner.

Future studies pertaining to the use of SP methodology online can examine: (a) the impact of virtual SPs on the learners’ affective domain-including contextual challenges and psychological, social, and cultural barriers; (b) the impact of virtual SPs on the learners’ ability to perform physical examinations using virtual SPs; (c) protocols for replacing clinical placement opportunities using the virtual SP approach and supporting instructors in the development of virtual scenarios; (d) barriers to engagement with online platforms, such as fear of technology, inability to see non-verbal cues, and the loss of physical touch and physical presence; and (e) how to compensate for these barriers in a virtual setting.

Since the pilot workshop was intended for instructors, identifying challenges allows us to further refine the workshop to meet the needs of instructors interested in embedding virtual SP methodology in their programs. We plan to continue our examination to learn more about changes to the learners’ attitudinal development towards SPs; their behaviours when using virtual versus face-to-face encounters and approaches; how these SP virtual-learning opportunities have impacted learners’ skills; and how these skills are being transferred to actual practicum or workplace settings.

## Conclusions

The virtual workshop, which reported high participant satisfaction, laid a foundation for subsequent iterations; indeed, plans are already under discussion to provide future workshops and to further develop the complexity and scope of this ambitious project for use across a variety of disciplines. This protocol dovetails with current conditions, as both the pandemic and the acceleration of integration of information technology are contributing to a rapid worldwide shift to online learning. We hypothesize that the virtual SP experience may contribute to sustained growth in online learning and that embedding virtual SPs will provide opportunities both for fostering student success and for enabling curriculum synergies. Engaging students through virtual scenarios that simulate the realities and complexities of practice and contribute to students’ success in their transition to the workplace is imperative during these unprecedented times.

## Figures and Tables

**Figure 5:**
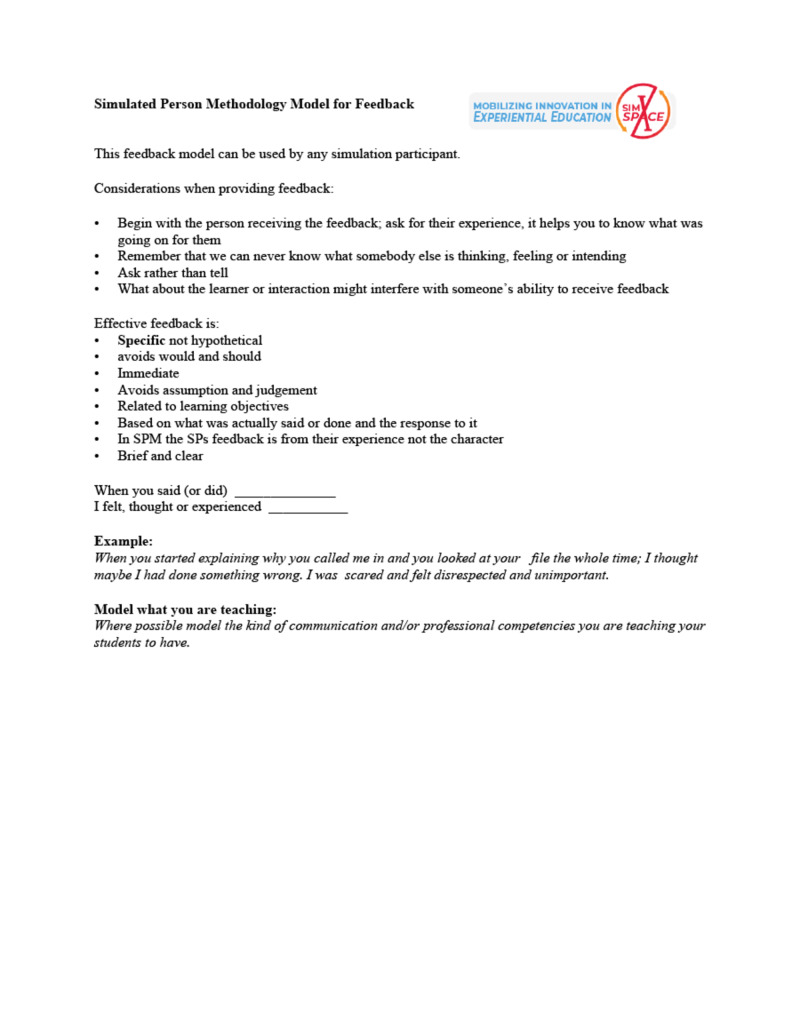
Simulated person methodology model for feedback

**Figure 6:**
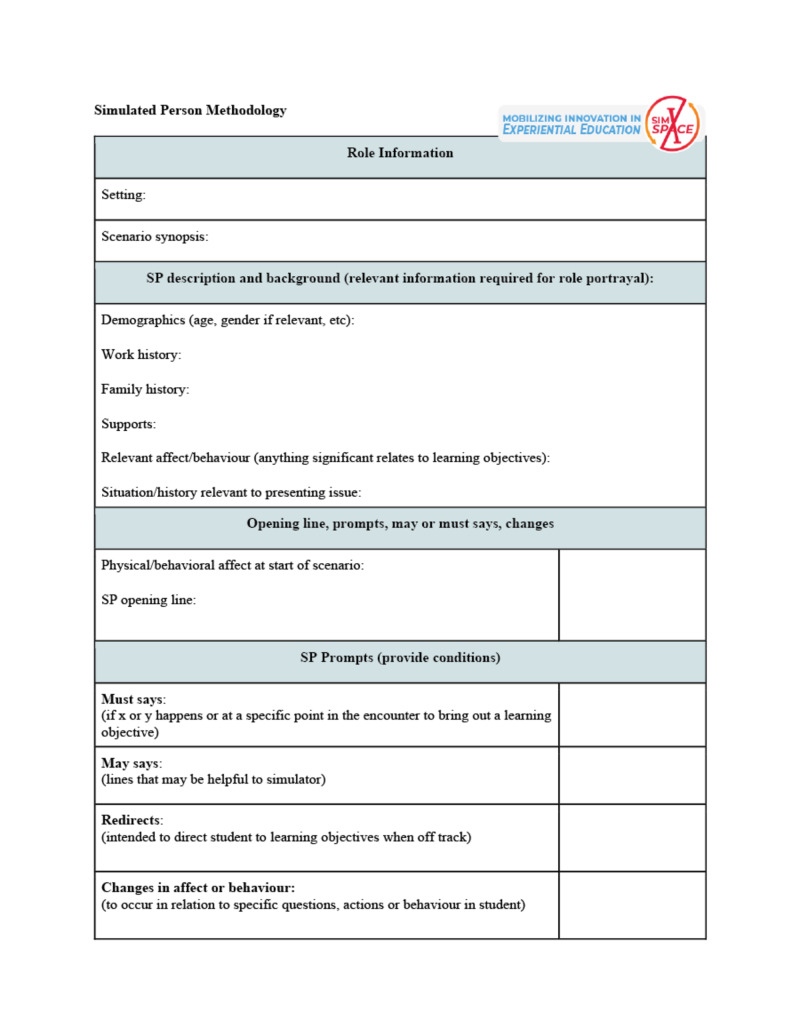
Template simulated person methodology

## Appendices

Appendix 1 

Appendix 2 
